# The innate immune axis drives aortic dissection pathogenesis through inflammation and presents novel therapeutic targets

**DOI:** 10.3389/fimmu.2025.1654622

**Published:** 2025-09-16

**Authors:** Can Xu, Wenping Chen, Xinyu Nie, Rui Xu, Xingyue Feng, Zhifen Chen, Dongjin Wang

**Affiliations:** ^1^ Department of Cardiac Surgery, Nanjing Drum Tower Hospital, The Affiliated Hospital of Nanjing University Medical School, Nanjing, China; ^2^ Institute of Cardiothoracic Vascular Disease, Nanjing University, Nanjing, China; ^3^ Department of Radiology, Nanjing Drum Tower Hospital, The Affiliated Hospital of Nanjing University Medical School, Nanjing, China; ^4^ Nanjing University Medical School, Nanjing, China; ^5^ Department of Cardiology, German Heart Centre Munich, Technical University of Munich, Munich, Germany; ^6^ Deutsches Zentrum für Herz- und Kreislaufforschung (DZHK), Partner Site Munich Heart Alliance, Munich, Germany

**Keywords:** acute aortic dissection, macrophage polarization, neutrophil, inflammation, extracellular matrix, cytokines

## Abstract

Acute aortic dissection (AAD) is a life-threatening cardiovascular emergency characterized by aortic layer separation and false lumen formation, with high mortality rates. Emerging evidence highlights the critical role of innate immunity in AD pathogenesis. Innate immune activation drives AAD progression through multiple mechanisms, including macrophage polarization (M1/M2 imbalance), neutrophil extracellular trap (NET) formation, and inflammasome activation. These processes amplify vascular inflammation via cytokine storms (IL-1β, IL-6, TNF-α) and oxidative stress, further promoting matrix metalloproteinase activation and smooth muscle cell phenotypic switching. The cGAS-STING pathway, triggered by mitochondrial DNA release, and TLR signaling act as central hubs connecting vascular injury to innate immune responses. This review synthesizes recent advances in the molecular mechanisms of AAD, focusing on aortic wall structural alterations, dysregulated signaling pathway, including TGF-β, Ang II, STING, and TLR cascades, and immune-inflammatory responses mediated by innate immune components. A deeper understanding of these innate immune components may lead to improved diagnostic biomarkers and targeted therapies for AAD management.

## 1 Introduction

Acute aortic dissection (AAD) is a fatal cardiovascular emergency in which blood penetrates a tear in the intima, creating a false lumen within the medial layer. AAD shows distinct age- and sex-specific epidemiological patterns and carries a very high mortality rate. Population studies indicate that AAD occurs predominantly in men aged 60–70 years (1). Clinically, patients typically present with abrupt, severe tearing or knife-like chest or back pain, often complicated by aortic valve insufficiency, acute heart failure, or myocardial infarction (2). The defining pathophysiological event is intimal disruption under hemodynamic stress, which separates the intima from the media, generating a false lumen alongside the true lumen.

Although the molecular drivers of AAD remain incompletely defined, several mechanisms have been implicated, including endothelial-to-mesenchymal transition (EndMT) (3), phenotypic modulation of medial smooth muscle cells (SMCs) (4), fragmentation of medial elastic fibers (5), extracellular matrix degradation in the adventitia, and vascular inflammation (6). Recognized high-risk factors for AAD encompass hypertension, advanced age, obesity, tobacco use, and genetic predisposition (7). Elucidating the molecular basis of AAD is essential for advancing strategies for prevention, early detection, and therapy. This review summarizes recent insights into AAD pathogenesis from five perspectives: aortic wall structure and cell biology, matrix metabolism, inflammation, oxidative stress, and associated signaling pathways.

## 2 Structural alterations of the aortic wall underlie the pathogenesis of AD

### 2.1 Injury to the intima and media and degradation of the extracellular matrix

The aortic wall comprises three layers, including intima, media, and adventitia, composed of vascular cells and extracellular matrix (ECM) components undergoing constant remodeling to maintain biomechanical integrity. Disruption of this balance initiates AAD. The intima, lined by endothelial cells, acts as a barrier and regulator of vascular homeostasis. In AAD, hypertension disrupts endothelial tight junctions, promoting macrophage infiltration and inflammation, leading to intimal rupture (8). The media contains SMCs embedded in elastic fibers, collagen, and proteoglycans. Medial degeneration—marked by SMC loss and elastic fiber fragmentation—is implicated in AAD onset (9). Fibrillin-1, crucial for microfibril structure and TGF-β regulation, when mutated, disrupts SMCs and promotes matrix degradation and inflammation (7, 10). EMILIN-1, essential for elastic fiber assembly and suppression of TGF-β activity, when deficient, induces fiber disarray (11). Collagens I and III, determinants of stiffness and elasticity, are aberrantly upregulated in AAD, where excessive deposition promotes fibrosis and vascular rigidity (12). The ECM provides structural and signaling support, regulating tensile strength and cell behavior. Dysregulated ECM turnover impairs vascular integrity. Matrix metalloproteinases (MMPs), especially MMP-2 and MMP-9, are elevated in AAD, driving ECM degradation (13, 14). Tissue inhibitors of metalloproteinases (TIMPs) maintain MMP balance; their dysregulation results in pathological remodeling (15). Thus, ECM imbalance and proteolytic activity facilitate AAD pathogenesis.

### 2.2 Activation of ECM remodeling pathways

Transforming growth factor-beta (TGF-β), encompassing TGF-β1, -β2, and -β3, orchestrates proliferation, differentiation, apoptosis, ECM synthesis, and motility (16). TGF-β1 and -β3 engage type II receptors, which activate type I receptors to initiate canonical Smad signaling: R-Smads are phosphorylated, form complexes with Smad4, and translocate into the nucleus to regulate transcription, with inhibitory Smads maintaining homeostasis (17, 18). TGF-β is indispensable for early aortic development and ECM homeostasis, yet in AAD it is aberrantly activated, driving ECM degradation through MMP-2 and MMP-9. Neutralizing TGF-β antibodies prevent AAD initiation (10), but genetic ablation of pathway components fails to mitigate disease, and mutations in TGF-β signaling genes are frequently identified in AAD patients (19). These paradoxes suggest that physiological TGF-β is vital for aortic integrity, whereas its hyperactivation contributes to AAD pathology. Ang II, the principal effector of the renin–angiotensin system, constitutes another critical signaling axis intricately linked to TGF-β signaling. Beyond its canonical roles in vasoconstriction, sodium reabsorption, and aldosterone synthesis, Ang II induces adhesion molecules, cytokines, chemokines, and pro-fibrotic mediators, thereby promoting vascular inflammation (20). Notably, Ang II potentiates TGF-β signaling via the angiotensin II type 1 receptor (AT1R) (21). In *ApoE* ^-^/^-^ mice, sustained Ang II infusion provokes elastin degradation and inflammatory mediator release, culminating in AAD (22). Ang II also governs inflammatory initiation by engaging integrins and vascular endothelial (VE)-cadherins, enhancing vascular permeability and leukocyte infiltration, whereas Ang II deficiency delays inflammatory activation (23). Thus, dysregulated Ang II destabilizes vascular homeostasis. Its elevation across cardiovascular diseases highlights its utility as a diagnostic biomarker and its indispensability in modeling vascular pathology.

## 3 Inflammatory cell infiltration and immune responses

### 3.1 Inflammatory cell subsets

#### 3.1.1 Macrophages

Macrophages, as central phagocytes of the innate immune system, play a central role in the pathogenesis of AAD by mediating inflammation and presenting antigens to initiate adaptive responses (6). They are broadly classified into pro-inflammatory M1 and anti-inflammatory M2 subsets. In AAD lesions, M1 macrophages predominate, driven by STAT and NF-κB signaling cascades regulated by miR-720 and miR-127. This polarization promotes the release of TNF-α, ROS, IL-1, IL-6, and NO, while suppressing IL-12, IL-23, and IL-10, thereby amplifying vascular injury (24, 25). During early AAD stages, M1 macrophages infiltrate from the adventitia into the media, initiating extracellular matrix degradation (6), whereas M2 macrophages emerge later to support repair. Macrophage-derived MMPs and interleukins sustain inflammatory loops via cytokine signaling (26). Moreover, angiotensin II exacerbates macrophage recruitment and activation through the KLF6–GM-CSF axis, upregulating MMPs and ADAMTS-1 expression, which collectively compromise aortic wall integrity (27).

#### 3.1.2 Neutrophils

**Figure 1:**
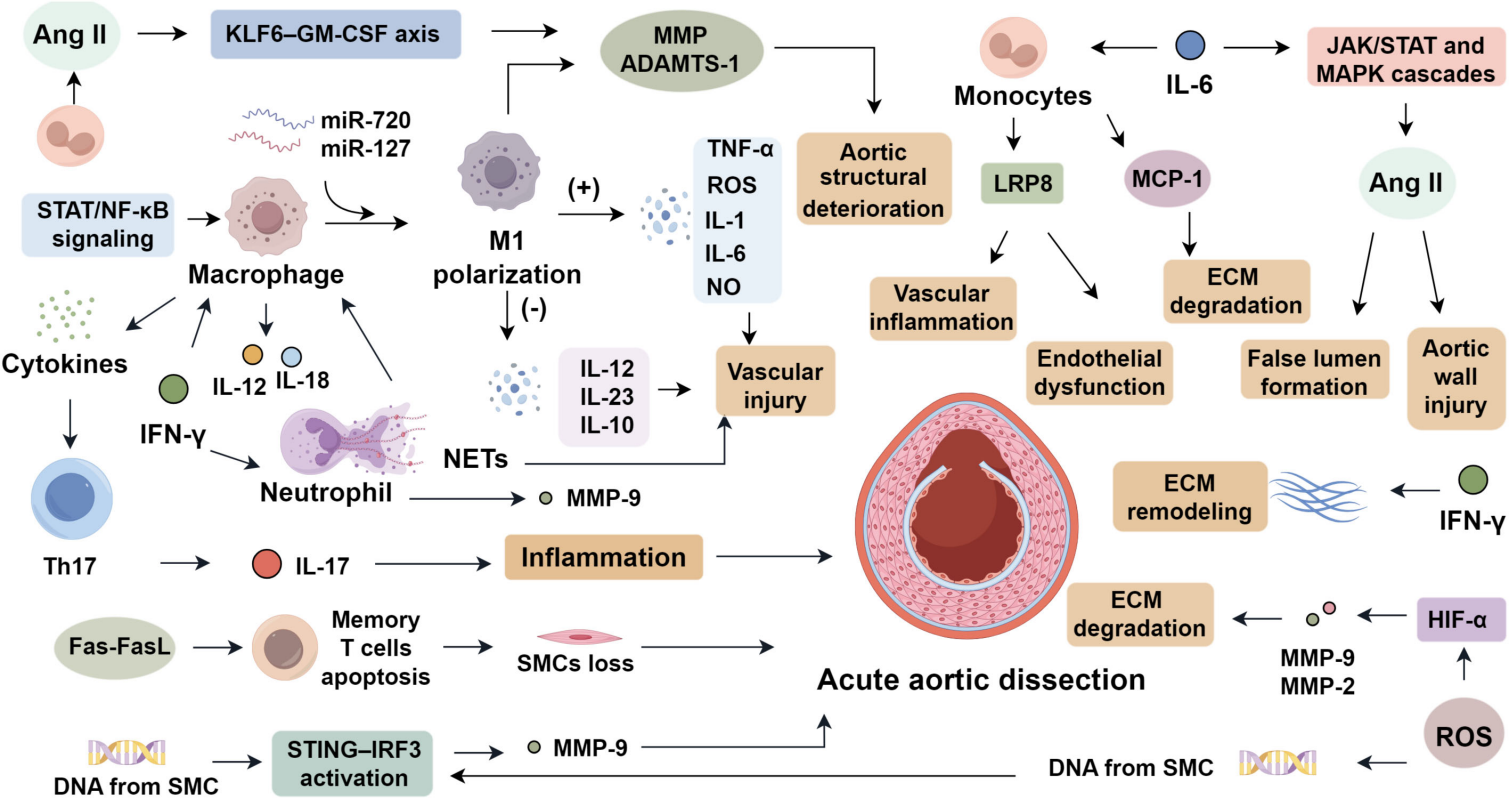
Innate immune in aortic dissection pathogenesis.

Neutrophils initiate acute inflammation by rapidly infiltrating injured sites and orchestrating secondary immune recruitment via cytokine release, thus forming the frontline defense barrier (28). In AAD, activated neutrophils contribute to both inflammatory injury and maladaptive tissue remodeling (4). Neutrophil extracellular traps (NETs) are formed through a process known as NETosis, which is triggered by the generation of reactive oxygen species (ROS), calcium influx, and activation of peptidylarginine deiminase 4 (PAD4), leading to chromatin decondensation and histone citrullination (29–31). NET-derived components such as neutrophil elastase (NE), myeloperoxidase (MPO), and citrullinated histones exert cytotoxic effects on vascular smooth muscle cells (VSMCs) by disrupting cell membrane integrity, promoting apoptosis, and inducing phenotypic switching toward a synthetic, matrix-degrading phenotype (32–34). In macrophages, these NET components enhance inflammasome activation, upregulate pro-inflammatory cytokines, and perpetuate the inflammatory cycle within the aortic wall (35, 36). NET accumulation in AAD correlates with disease severity, promotes macrophage cytokine secretion, and facilitates Th17 polarization (4, 37). NET-associated components, such as NE and its target TBL1x, facilitate inflammatory cell migration and vascular smooth muscle cell phenotypic switching (5). Clinical studies reveal elevated granzyme and NET markers in aortic tissues of AAD patients, with circulating NET levels linked to in-hospital mortality and one-year survival (4). In experimental models, neutrophil depletion or inhibition of vascular infiltration reduces MMP expression and significantly decreases AAD incidence (38), underscoring neutrophils and NETs as potential therapeutic targets (**Figure 1**).

#### 3.1.3 T lymphocytes

T lymphocyte activation plays a critical role in the pathogenesis of AAD. Elevated levels of CD3^+^, CD4^+^, CD8^+^, and CD45^+^ T cells have been identified in the aortic wall of AAD patients, indicating active local immune involvement (39). Within T helper subsets, Th1, Th9, Th17, and Th22 cells and their transcriptional regulators are obviously upregulated, while Th2 and Tregs are downregulated, suggesting a protective role for the latter (40). Th17 and Tregs originate from a shared precursor and are both dependent on TGF-β; however, IL-6 promotes Th17 differentiation at the expense of Tregs (41). Elevated IL-6 and Th17-derived IL-17 levels have been confirmed in AAD tissues (42), supporting the therapeutic potential of IL-6 inhibition. IL-17 promotes extracellular matrix (ECM) degradation by stimulating MMP-2 and MMP-9 expression in VSMCs and macrophages (42–45), weakening the structural integrity of the aortic wall by degrading elastin and collagen (46, 47). Besides, IL-17 stimulates the secretion of pro-inflammatory cytokines and chemokines (IL-1β, TNF-α, CCL2), further enhancing immune cell recruitment and inflammation within the aortic media (48–50). The IL-6/STAT3 axis acts as a key amplifier in this cascade. IL-6 binding to gp130 activates JAKs, driving STAT3 phosphorylation and nuclear localization, thereby enhancing transcription of RORγt, the master regulator of Th17 lineage commitment and IL-17 production (51–54). STAT3 activation also promotes MMP expression in vascular cells, intensifying ECM degradation. This feedforward loop—linking IL-6, STAT3, and IL-17—potentiates vascular inflammation, smooth muscle cell phenotypic switching, and structural disintegration, hallmarks of AAD (55). Tregs, though typically anti-inflammatory via IL-10 production, also exhibit functional heterogeneity; increased CD25^+^ Tregs have been linked to vascular inflammation in carotid artery disease (56, 57). Additionally, Fas–FasL-mediated apoptosis of memory T cells may induce smooth muscle cell loss and vascular wall weakening, exacerbating AAD risk (58).

#### 3.1.4 Monocytes

Monocytes, circulating components of the adaptive immune system, possess phagocytic capacity and activate other immune cells. Based on surface markers, they are classified into three subsets: classical (CD14^++^CD16^−^), intermediate (CD14^++^CD16^+^), and non-classical (CD14^+^CD16^+^) (59). Among these, classical monocytes exhibit the highest pro-inflammatory potential, characterized by robust phagocytic activity and the capacity to secrete cytokines such as TNF-α and IL-1β upon activation (60). These cells are rapidly recruited to sites of vascular injury and are primarily responsible for initiating and amplifying inflammatory responses in the early phase of AAD (61, 62). In AAD, classical monocytes are markedly elevated, while intermediate subsets are reduced. Activated monocytes interact with platelet glycoprotein Ibα (GPIbα) and coagulation factor XI (FXI) to promote local thrombin generation, thereby exacerbating vascular inflammation and hypertension, and accelerating AAD progression (63). Haider et al. (64) demonstrated that IL-14 stimulation reduces NF-κB p65 phosphorylation and decreases monocyte apoptosis *in vitro*, suggesting that beyond promoting macrophage differentiation, the NF-κB pathway may also support monocyte survival and differentiation. Monocytes also release low-density lipoprotein receptor-related protein 8 (LRP8), which triggers vascular inflammation and endothelial dysfunction, further implicating them in the pathogenesis of AAD (65). These findings imply that monocytes may also play a contributory role in Ang II–mediated AAD development.

### 3.2 Inflammatory factors

#### 3.2.1 Cytokine and chemokine

The interleukin-6 (IL-6) cytokine family includes IL-6, IL-11, IL-30, IL-31, and non-IL molecules, primarily secreted by lymphocytes, monocytes/macrophages, adipocytes, tumor cells, and endothelial cells (66). Elevated IL-6 levels are observed in patients with AAD compared to healthy controls (67). Sano and Anzai (68) found that chemokine-dependent signaling induces neutrophilia and infiltration into the dissected aorta, where IL-6 contributes to aortic dilation and rupture. Tieu et al. (69) noted IL-6’s localization in the adventitia, promoting monocyte recruitment, MCP-1 secretion, vascular inflammation, and ECM degradation. The IL-6 signaling pathway involves gp130, which recruits co-receptors like the leukemia inhibitory factor (LIF) receptor and oncostatin M (OSM) receptor, activating JAK/STAT and MAPK cascades (70). These pathways enhance angiotensin II (Ang II) signaling, causing vasoconstriction via the renin-angiotensin system (RAS), elevating blood pressure and exacerbating aortic wall injury, leading to intimal tearing and false lumen formation (71). Chemokines are small proteins that guide the directional migration of cells through interactions with G protein–coupled receptors on target cells. Based on N-terminal cysteine residue arrangement, they are classified into five subfamilies: CXC, CX, CC, XC, and CX3C (72). Most studies focus on the inflammatory roles of the CXC and CC families. The CC family includes ~28 members, such as CCL2 (MCP-1), which enhances IL-6 expression and reduces macrophage apoptosis in aortic walls, mitigating Ang II–induced AAD progression (69). CCL3 (MIP-1β) has been linked to AAD, with elevated levels found in AAD patients (73). The CXC family includes ~17 members, such as CXCL1, which is upregulated in AAD and promotes neutrophil infiltration and IL-6 expression, leading to aortic dilation and rupture (74). CXCL4 exacerbates atherosclerosis by enhancing TLR2 signaling and lipid deposition at the aortic root. These findings highlight chemokines as critical mediators in vascular inflammation, warranting further research into their roles.

#### 3.2.2 Tumor necrosis factor-α and interferons

The tumor necrosis factor (TNF) superfamily includes over 20 members, such as TNF-α, B cell activating factor (BAF), photosensitizer-β (PS-β), TNF-related apoptosis-inducing ligand (TRAIL), and receptor activator of nuclear factor kappa-B ligand (RANKL), all of which potentiate inflammation through NF-κB signaling. Liu et al. (75) reported significantly increased serum TNF-α levels in AAD patients. TNF-α is also implicated in the regulation of vascular SMC apoptosis, a process central to AAD progression (76). Notably, the concentration of TNFα varies with disease stage and progression (77), suggesting temporal heterogeneity in its regulatory mechanisms and indicating the need for time-resolved studies. Interferons (IFNs), a class of cytokines with potent antiviral, antiproliferative, and immunomodulatory properties, are crucial components of innate immunity (78). IFNs are classified into type I, II, and III. Type I IFNs include IFNα, IFNβ, IFNδ, IFNϵ, and IFNκ, which exert broad antiviral and antiproliferative functions (79). Type II IFN (IFN-γ) is produced by T lymphocytes, antigen-presenting cells, and NK cells, and exerts immunoregulatory functions (80). Type III IFNs include IFN-λ1 (IL-29), IFN-λ2 (IL-28A), IFN-λ3 (IL-28B), and IFN-λ4. Among these, IFN-γ plays a central role in AAD progression. It stimulates macrophages to secrete IL-12 and IL-18, which further activate the NF-κB pathway, establishing a positive feedback loop that enhances IFN-γ expression (81). It also modifies the extracellular matrix (ECM) by enhancing neutrophil infiltration and stimulating MMP9 release. In aortic SMCs, IFNγ activates JNK signaling, leading to cJun phosphorylation and MMP2 upregulation (82). Furthermore, IFNγ contributes to SMC phenotypic switching, as evidenced by its ability to suppress contractile markers such as SM22α and calmodulin (83). Collectively, these findings support IFN-γ’s role in exacerbating vascular inflammation by modulating ECM remodeling and SMC plasticity.

### 3.3 Activation of inflammation-associated signaling pathways

The stimulator of interferon genes (STING) pathway, a key component of the cytosolic DNA-sensing cGAS–STING axis, is a critical mediator of pro-inflammatory responses (84). It detects intracellular pathogenic DNA and initiates immune signaling cascades that contribute to tissue inflammation and injury (85). Cytosolic DNA activates cyclic GMP–AMP synthase (cGAS), which generates the second messenger cGAMP to activate STING. Activated STING recruits and activates kinases such as tank-binding kinase 1 (TBK1), leading to phosphorylation and activation of downstream effectors including interferon regulatory factor 3 (IRF3) and NF-κB (86). These pathways drive pro-inflammatory gene expression and apoptosis. In vascular injury, damage-associated DNA from SMC nuclei or mitochondria is phagocytosed by macrophages, resulting in aberrant STING–IRF3 activation and increased MMP-9 expression, thereby contributing to AAD development (87). Toll-like receptors (TLRs) are pattern recognition receptors that sense pathogen-associated molecular patterns and initiate innate immune responses. TLR signaling is mediated via the MyD88-dependent and TRIF-dependent pathways (88). MyD88 recruits IRAK-4 to TLRs through death domain interactions, initiating IRAK-1 phosphorylation and subsequent activation of TNF receptor–associated factor 6 (TRAF6). This leads to dual activation of MAPKs via AP-1 and the TAK1–TAB–NF-κB complex. The TRIF-dependent pathway, primarily activated by TLR3 and TLR4, promotes TBK1-mediated IRF3 phosphorylation and type I IFN expression. TRIF also facilitates NF-κB activation through interactions with RIP1 (89). Elevated TLR4 expression has been observed in AAD patients (90), implicating its role in vascular inflammation and remodeling. Oxidative stress, driven by the accumulation of reactive oxygen species (ROS), is a major contributor to AAD initiation and progression. In the presence of Ang II, endothelial NADPH oxidase 2 (Nox2)–derived ROS stimulates cyclophilin A (CyPA) secretion, which in turn activates MMPs and ROS production in SMCs (91). Increased aortic stiffness—a precursor to AAD—induces mitochondrial dysfunction in SMCs, exacerbating ROS generation and promoting a phenotypic shift from contractile to synthetic states (92, 93). ROS further upregulate hypoxia-inducible factor-1α (HIF-1α), which enhances MMP-2 and MMP-9 expression and accelerates ECM degradation (94), facilitating AAD onset.

### 3.4 Crosstalk among signaling pathways in AAD

Multiple signaling pathways, including TGF-β, Ang II, STING, and TLR cascades, intersect to drive the complex pathogenesis of AAD. TRAF6 acts as a central adaptor, linking TGF-β receptor activation to downstream effectors such as TAK1, p38 MAPK, NF-κB, and JNK (95), while also being engaged by TLR signaling. Mitochondrial damage caused by ROS leads to the release of cytosolic DNA, which activates the STING–TBK1–IRF3 pathway in aortic smooth muscle cells, resulting in pro-inflammatory responses and cell death (87, 96). STING and TLR pathways share several common regulatory nodes; for instance, TRIF is essential for STING dimerization and downstream signal propagation (97), while STING activation induces SOCS1, which negatively regulates MyD88 and thereby modulates TLR signaling intensity (98). Moreover, IRF3, downstream of both STING and TLR pathways, can bind Smad3, thereby preventing its recruitment to TGF-β receptor I and dampening TGF-β–mediated transcription (99). These intersecting pathways collectively contribute to extracellular matrix degradation, oxidative stress, immune cell infiltration, and phenotypic modulation of vascular smooth muscle cells. While TGF-β primarily governs matrix remodeling and structural integrity, TLR signaling intensifies the inflammatory response. Despite the convergence on shared effectors such as NF-κB, each pathway exerts distinct biological functions. A deeper understanding of this integrated signaling network is crucial for identifying novel diagnostic markers and therapeutic targets for the prevention and treatment of AAD.

## 4 Conclusion

Acute aortic dissection is a life-threatening vascular disorder driven by innate immune activation, inflammatory cascades, and structural degradation of the aortic wall. Endothelial injury, smooth muscle cell dysfunction, and extracellular matrix breakdown create a vulnerable microenvironment, while dysregulated TGF-β, Ang II, and TLR-STING pathways amplify inflammation and oxidative stress. Innate immunity plays a pivotal role, where macrophage polarization imbalance, neutrophil NETosis, and monocyte-derived cytokines exacerbate vascular injury. Additionally, cGAS-STING and TLR signaling propagate DNA damage responses and matrix metalloproteinase activation.

The crosstalk between these pathways creates a vicious cycle of ECM degradation and smooth muscle cell phenotypic switching. For instance, ROS-induced mitochondrial DNA release fuels STING-dependent inflammation, further aggravating disease progression. Therapeutic strategies targeting innate immunity, such as NET inhibition, IL-6 and IL-1β blockade, or STING antagonists, may complement conventional approaches by mitigating early inflammatory triggers. Future research should prioritize biomarkers of innate immune activation for early diagnosis, as well as immunomodulatory therapies to disrupt pathogenic feedback loops. By integrating mechanistic insights into innate immunity with vascular biology, precision interventions could significantly improve outcomes in this high-mortality condition.
